# Why the way we consider the body matters – Reflections on four bioethical perspectives on the human body

**DOI:** 10.1186/1747-5341-2-30

**Published:** 2007-12-04

**Authors:** Silke Schicktanz

**Affiliations:** 1 University of Göttingen, Dept. of Medical Ethics and History of Medicine, Humboldtallee 36, 37073 Göttingen, Germany

## Abstract

**Background:**

Within the context of applied bioethical reasoning, various conceptions of the human body are focused upon by the author in relation to normative notions of autonomy.

**Results:**

The author begins by descriptively exploring some main positions in bioethics from which the "body" is conceptualized. Such positions conflict: the body is that which is constitutive of the individual's experience and perception, or it is conceived of materially or mechanistically; or as a constructed locus, always historically and culturally transformed. The author goes on to suggest a methodological approach that dialectically considers embodiment from four different perspectives: as bodily self-determination, as respect for the bodily unavailability of the other, as care for bodily individuality; and lastly, as acknowledgement of bodily-constituted communities. These four perspectives encompass autonomy in two of its main interpretations: as the capability of a person to act independent of external forces, and as the moral ideal of pursuing individual wishes by means of role distance, self-limitation and universalization. Various bioethical cases are utilized to show how the four perspectives on the body can complement one another.

**Conclusion:**

The way we consider the body matters. The author's dialectical method allows a premise-critical identification and exploration of bioethical problems concerning the body. The method is potentially applicable to other bioethical problems.

## Introduction

During the 1970s, a number of performance artists shocked the public by making their bodies the subject of artistic performances. By being thus displayed, the body itself becomes both the medium of the artistic work and the scene on which it takes place. In the performance Zerreißprobe (1970) the Austrian artist Günter Brus injured himself by cutting his head and thigh with a razor blade. The vulnerability of the flesh was to be shown by means of the extreme display of a body disfigured by pain and by interventions from the outside. Brus' perfomance at the same time was intended to demonstrate limits and extremes. The American Chris Burden had his left arm shot by a friend in the course of the performance Shoot (1971), though the focus here was less on the vulnerability of the body than on the examination of ideals of masculinity and insensitiveness to pain as a test of courage. The French artist Orlan has been causing sensation since the 1990s by describing her body as "software" and declaring surgical operations on her face to be "art made of flesh and blood". In the course of these operations she regards herself as a living sculpture and "takes the liberty to experiment with her own body" [1].

In the 1970s, the liberty to be in charge of one's own body was discussed in another, quite different context as well: under the slogan "My body belongs to me!", thousands of women took to the streets in Germany, Britain, and the United States to demand a liberalization of the then existing abortion laws. These concerns and procedures – different as they may appear at first glance – point to the same important problem, namely the unclear or questionable relationships between the body, the self-determination of one and the same person, and its public articulation: What am I allowed to do with my own body, and to what extent can I permit others to do 'whatever they like' with their bodies?

Emerging from these public discussions of the 1970s, we may ask whether these concerns and questions are still relevant for recent bioethical debates. Both examples, modern performance art and political demonstrations, have pointed to the political and social dimension of these questions, as especially 'external' power over the body was criticized. However, in the early 1970s many people did not understand these primarily as ethical *questions*. It was for many deemed as self-evident that the body is an object of self-determination and action. Bioethics itself, understood as the systematic consideration of ethical problems and ethical judgments on the basis of rational argumentation, was in its infancy at this time [2]. It was only in the 1990s that many scholars started to criticize the neglect of the body in academic bioethics (e.g. [3-5]). Additionally, in recent years, a "body boom" in media studies, history and social science has occurred. According to Anne Witz [6] the "corporeal turn" in sociology and feminism has emerged from a critique of the exclusion of certain bodies (such as women, disabled persons or elderly people) from the academic discourse. These should now no longer be neglected.

Additionally, one can call into question the tendencies of both analytical metaethics and also moral philosophy, as both center around notions of 'personhood', 'rationality', 'preferences' and 'self-determination' which are mainly conceptualized without any relation to the body, although bioethics often deals with problematic cases in which entities lack rationality and specific mental capacities, for example embryos, brain-dead patients, animals and so on. Thus, Margrit Shildrick [7] critically remarks that "bioethics is out of touch ... with bodies themselves, in the phenomenological sense in which the being, or rather the becoming, of the self is always intricately interwoven with the fabric of the body." (p. 1f).

Of course, international academic bioethics has itself developed into a multifaceted discipline, with mutual relationships between moral philosophy, sociology of science and clinical ethics. Thus, generalizations are always problematic. Nevertheless, I think it is not totally wrong to state that many scholars in applied ethics and bioethics still tend toward – as Shildrick calls them – 'conventional', positions which stress "fixed standards of judgement" [7] (p. 3). One of these standards is the value of autonomy and self-determination. Another common strand often favored by partisans of liberal self-determination sees the human body as an 'object' and as 'property' subject to personal, self-determined disposal. For example, the moral claim that "Every person should decide for themselves whether they want to donate their organ" is built upon the assumption that organ donation should be decided on by the donor themselves, seeing the body as 'property' or as a 'material object'. In contrast, postmodernism, or as Shildrick [7] puts it, postconventional ethics, sees the body as "leaky, uncontained, and uncontainable" (p. 7). From this vantage, the body is neither separable from the self nor from other embodied selves (p. 6). Many postmodernists also criticize the idea of thinking about the body as property, as an economic value, or as an instrument. (see e.g. [8,9])

Precisely in the field of body modification and bioethics we observe a clash of perspectives – in two ways. In the first place, there is a serious difference in the normative way of ethical judgment; secondly, there is a difference in how the body and embodiment are addressed. This distinction between mainstream bioethics and postconventional sociology and ethics could be difficult to overcome as long as both insist upon their "rightness".

However, in order to understand bioethics in a broader sense, as an academic discipline, sweeping aside for one moment social and political power plays in the academic world, it should be a basic interest for each and everyone to hear and to understand what the others are saying. This could be partly achieved by choosing a method of ethical reasoning which is open for the various, and sometimes conflicting views, and this paper is a first attempt at presenting such a method. It is hereby necessary to state that according to my understanding, normative bioethics is a *systematic, processual *method of ethical judgment (see [10]). It includes the description of an ethical problem, the analysis of underlying terms and opinions in the light of theories and practical experience, and finally an ethical evaluation or a recommendation on how to act. (Whether this is only true for problem-solving ethics or also for moral philosophy in a general sense I cannot discuss here). In this paper, I focus mainly on the issue of "problem definition"; an issue that is necessarily crucial for all kinds of moral analyses and for final evaluation. This understanding of bioethics is applicable to 'conventional' deontological, utilitarian and postconventional (such as care-ethics) perspectives. (What matters for each distinct position are the following decisions: Who do we identify as the relevant actors? What are the relevant values? And finally: What is our justification for them?). Thus, the aim of this paper is not to discuss postmodernism itself. Instead, I intend to critically reflect on mainstream medical ethics (which I see myself as a part of) and I want to show why and where some of the postmodern observations are very important and helpful.

In the first section, I want to show that the socio-historical and phenomenological approach on the one hand and 'conventional' bioethics on the other cross each other (not only, but most prominently) in the debate about 'bodily limits' and 'transgressing body borders'. Conventional bioethics especially could profit from socio-historical and empirical-phenomenological investigations of these phenomena because they help clarify descriptive or anthropological premises about the 'body'. Hence, I want to argue that – independently of the way in which the body is described in bioethics, whether as material and distinct from mind or as dynamic and socially interconnected – we always deal with a value-laden phenomenon. Instead of seeking to avoid hidden moral assumptions, I suggest a methodological approach of making them explicit. By relating the contrary positions to one another dialectically, heuristic use can be made of the recent dichotomies in bioethics. I have chosen for this purpose 'autonomy', one of the central conceptions of contemporary U.S. American and Mid-European bioethics. A 'conception' means here an abstract notion or system of thoughts which is bundled in a term. However, such a term could be conceptualized in various ways. Thus, 'autonomy' covers several aspects of self-determination, such as the opportunity for free decision, but also the capacity of voluntary self-limitation [11]. I develop four different normative perspectives of how autonomy and embodiment could be interlinked. I suggest that these recent conceptions of bodily autonomy could complement one another, instead of our presupposing only 'one' right view. This allows, in my understanding, an improvement to bioethical normative reasoning, and also helps ethicists interested in concrete problem-solving start right from the beginning with a critical sensitivity to their own premises on what autonomy and the body 'mean'. In a third step, I want to provide an outlook on how my suggested method broadens our way of *asking *ethical questions by discussing briefly three examples chosen from the fields of transplantation medicine, neuroprosthesis and cosmetic surgery. The chosen examples should also show that the way we consider the body in bioethics is not only an issue relevant for women's health or reproductive medicine [12], but for all topics in bioethics. The aim of the approach is to be open to multi-dimensional categories in order to advance the identification and description of bioethical problems.

## Results

### Part 1: The Body in ethical, social and historical considerations of medicine

#### The body is more than the locus

Of course, the body has always been and will always be the physical object of medical interventions and biomedical innovations, and it is therefore already included in bioethical thought [3]. Within the medico-ethical canon of non-maleficience, of risk aversion, of healing and care, the body as soma/the body as physicality is always involved as a 'locus' (where the intervention or the action takes place). Eventually, the bioethical discussion's primary focus on the body happens in the context of the veto right to bodily integrity or as moral concerns about 'suffering', often understood as a physical state. Both foci feature a predominantly instrumental relation to the body, because the body is regarded as a carrier of, or vehicle for, the decisive wishes, preferences or interests of a person. The understanding of the body as socially or culturally constructed or negotiated plays no role either for the justification of veto rights or for the situation of physical suffering. For example, in the case of 'suffering', the search for physiological parameters and quasi-objective criteria to measure it (as is a hot topic in animal ethics) refers to the 'natural', materialistically-conceived body. This conception, which has been described as the 'absent body' [5], is based on the assumption that the generation and validity of wishes and interests can be analyzed on the basis of the physical body alone without reference to the body in its social and phenomenological meaning. According to Leder [13], this is due to the after-effects of Cartesian dualism and its materialist conception of the body as a machine. The human being and its personality were located exclusively within the bodiless spirit. But further contexts are also important. On the one hand, many writers mention the individual "constitutions of meaning qua the body" [14]. Embodiment is regarded as experienced body sensation, whereby the body is understood as the scene of the immediate, of the pre-reflexive or of life's taking place, in the context of individual actions, perceptions and experiences in their role for human self-understanding [15,13]. (The idea of embodiment must not be used interchangeably with the idea of naturalness, as especially the boundaries between nature and culture remain unclear with respect to the body.) According to the early phenomenological tradition of Max Scheler (1913) [16], the German language allows for a distinction between "Körper" and "Leib", which relates to the difference between "thing body" (or "flesh") and "lived body". This distinction highlights some Cartesian presumptions, but is not identical with the body-mind-distinction. Later, Merleau-Ponty [11] pointed rather to the ambiguity of the lived body as ["*corps propre*"] – an intermediate between flesh and the body as it is subjectively experienced by the mind.

On the other hand, scholars from history and social science stress the "historicity of the body". In this stance, we should pay more attention to the social and historical contingency and flexibility of the localization of perception, and of the description and disciplining of the body. The understanding of the body as socially constructed 'corporality' is interpreted as a historically and culturally relative variable [17,18]. Following Donna Haraway [19] body images are of linguistic nature and do not represent the real body but are in fact 'objects of knowledge'. However, a very radical socio-constructivist approach would eliminate this perspective on the body as well. The conception of a (totally) flexible and ambivalent body reduces the body to nothing, or to a mere space for projection. In postmodern transhumanism the body is often not ascribed a value of its own. Within the phenomenological approach, embodiment as an entity in its own right is seen as giving immediacy and materiality to individuals and societies, and as thus constitutive for human self-understanding. With this approach, the precarious nature of conceptualizing the body becomes obvious. Phenomenology points to the already implicit normative significance of the body and the discussions about what should be done with, and made of it [14]. Although one could fear that the loss of certainty concerning our body may result in a new form of absence of the body, the socio-cultural and poststructural criticism allows us to open our reasoning in further directions, such that we can now reflect on the one hand on the phenomenological perspective of perception and experience of embodiment and on the other hand on the perspective on the body as corporality that sees it as formed by culture, socialization or the history of science.

Since the goal of this article is to develop an approach which is open for different premises and perspectives regarding the body, I do not want to restrict my definition to one theoretical strand. Therefore, I suggest using the term "embodiment" to encompass the different perspectives.

#### Body limits as moral and epistemic uncertainties

What is of interest here is that both of the last mentioned approaches question the certainty of the claim that the body is only the physical locus of medical interventions on a theoretical level, while medicine and biotechnology question this certainty on a practical, everyday level. I suggest that the "body boom" continues because the time we live in chooses transgressions between bodies and also categories (in the sense of playing with limits) as a focal point for technical innovations and social designs of life (see also [7]). The body boom is a result of the experienced and conscious play with the limits of the body.

However, the reactions to this are quite ambivalent. Whereas some free such transgressions from taboos by describing them as a logical consequence of technological development [20], or even demand them, as the so called transhumanists do, others lament the (often hidden) increasing danger for both society and the individual posed by the new technical domination of the body and its perfection towards the elimination of finiteness [21].

From an ethical point of view it remains to be analyzed whether, for instance, our intuition is morally wrong that certain forms of utilization of the body are not permitted, and whether certain practices must necessarily be judged as a morally problematic instrumentalisation of the body. It is my thesis, though, that such an ethical analysis will have to consider the anthropological and epistemological premises that form the basis for the relationships between embodiment and normative values. The following three observations shall serve as an introduction to my considerations of the intertwining of ethical, anthropological and epistemological dimensions:

1. Certain biomedical procedures (amongst others transplantations and implantations) activate moral intuitions or discomfort more strongly than others do, and thus raise questions concerning the normative relevance of the body;

2. At the same time, technologies that transgress both borders and 'limits' question the traditional categories of order of the Western culture (influenced by the Judeo-Christian Tradition, the Enlightenment and scientific ideas since the nineteenth century) (see e.g. [22]). This pertains above all to the following binary categories:

- *Nature – culture*: this basic distinction, based on Aristotelian thinking, is blurred for example, in the case of the cultivation of cells or artificially produced organisms.

- *Human person – machine*: this distinction is challenged by manipulation of the mind through brain-implanted chips and brain-computer interfaces.

- *Human being – animal*: This Aristotelian and also Judeo-Christian distinction between humans and animals is questioned by, for example, the creation of human-animal-chimeras.

- *Internal – external*: The nineteenth century idea of physical and social boundaries is challenged for instance by questioning the ownership of an explanted organ or of an embryo created in vitro.

- *Body – mind*: the Cartesian distinction between the body as a machine and the mind as the ratio is challenged for instance through the transplantations of brain tissue.

3. There are different reactions to the questioning of these conventional orders:

- There is the naturalist argument, where the body is understood in a materialist way as irrelevant for the formation of norms. A 'value' of the body can only be established by referring to the interests or values of the 'users' of this particular body;

- There is a constructivist-relativist discussion about the variety of body conceptions, which either refuses all universally applicable claims to truth or, in a radical form, denies the body's materiality;

- A normative, prescriptive relevance of the body is postulated, making of it something resistant and unavailable, with a value of its own – an end in itself.

Let us consider the first observation. Research in the history of medicine and culture suggests that the development of modern medicine (starting with anatomy, physiology, cellular pathology, bacteriology and hygiene, human genetics) has successively turned the human body into an object, and then dissected, regionalized, localized and standardized it [23,24]. As a consequence, the body and its parts tend to be regarded through a view known as "empiricist materialism" ([3]: 300) and are seen as exchangeable and open to modification.

Transplantation medicine, for instance, is historically clearly based on the localization theory of illness that dates from the mid-nineteenth century [25]. However, the practice of transplantation medicine could only be established on the basis of insights resulting from systemic immunology in the second half of the twentieth century, including the knowledge of how to understand and manipulate several pathways of immunological rejection. For a long time, the ethical discussion of transplantation medicine neglected consideration of the transfer of organs with respect to its integration into the body image and the union of body and spirit, although there were many sociological and anthropological publications on these issues (see e.g. [26,27]) The premise of interchangeability as regards physicality and personal identity was only questioned in ethics against the background of discussions about the transplantation of neuronal tissue or even entire heads [28,29], whereas other body parts were not regarded as constitutive of identity. Such scientific and technological objectivation and fragmentation has been criticized as "de-bodiment of reality" and "ousting from perception the body itself" [30], and opposed as an attitude that exclusively focuses on control over the body. This criticism appears to contain the vague (and predominantly implicit) assumption that there exists a true or authentic perspective on the body-identity-relationship which one just needs to capture differently, in a new way [21].

Even if one cannot fully agree with this criticism, it nevertheless hints at a situation that I would classify as paradoxical: the mutual relationship between, on the one hand, still very prominent theoretical premises of objectification, fragmentation and blindness towards the (lived) body within everyday medical practice; and, on the other hand, socially and politically powerful critiques of increasingly dominating biotechnologies, which stress that the body is unique, must be perceived subjectively, and has independence and resistance.

The second observation concerns the ways in which such paradoxes or ambivalences are triggered. Traditional Western Occidental culture distinguishes very clearly between human being and machine. It characterizes the human being as a hybrid being that can be located between the two poles of 'nature' and 'culture', and it aims at the separation of the 'own' from the 'other' through individuation. According to my thesis, these poles are not only becoming blurred in the course of the biotechnological revolution, they are increasingly being dissolved. Certainly in our perception and language, mechanization, rationalization and instrumentalisation of the body or of living things increasingly moves the balance of the traditional order in one particular direction, predominantly towards *materialization *[30]. According to the sociologist Gesa Lindemann [31], certain medical technologies massively shake the hitherto common distinctions of relationships, namely the differentiation between the social interaction of two human agents on the one hand, and the relation of a personal agent and a non-personal object on the other. Lindemann demonstrates this by reference to her anthropological investigation of the ambivalent and sometimes contradictory attitudes of doctors, nurses and relatives to brain-dead patients in intensive care. Many bioethical problems result from this kind of situation: the question of how to treat and care for a brain-dead person in the clinical setting, whether it is permissible to remove organs (which will result in heart death) or whether others should care for months for a brain-dead pregnant woman so that the baby may grow and be brought to term. Conceptual difficulties, already present in identifying and evaluating the distinction between two human agents or a personal agent and a non-personal object, became obvious in discussions about the moral status of entities that transgress borders, for instance hybrid beings such as so-called chimeras. Such transitions from a loss of order towards normative evaluations lead to the third observation concerning reactions to such loss. The philosopher Hilge Landweer [32] has asserted that there are three very different and partly opposing strategies to place basic anthropological assumptions within contemporary images of the world:

a) Through the naturalist approach, the human being is reduced to a body and understood as a creature that is determined by physico-chemical processes beyond its control. Consequently, a person's self-relation would be nothing more than a complex neurophysiological process which could be changed and manipulated accordingly.

b) In the constructivist or postconventionalist approach, the essence of the human being can only be explained through historical and cultural discourses and contexts. Objective descriptions of the 'body' are no longer possible. There are only provisional 'truths' (see [7]: 5). And their analysis is reduced to the description of a series of conflicting and dispersed discourses. At last, the materiality of the body could be understood as a discourse, itself, depending on narrations of the 'body'.

c) In the "transformation"-approach, the materiality of the body is assumed, yet embodiment is seen as inaccessible for and through science. The body (as the sensual access/interface to the world) is understood as a precondition of all experience and knowledge. The body's independence and autonomy are defended. Despite all historical and cultural qualifications, this approach does not entirely neglect universally- applicable statements. However, the ways in which the body precedes all experience and knowledge cannot be captured by the terms 'nature' or 'biology', but escape any direct analysis.

My first interim conclusion is that all three positions are needed to explain how the normative relevance of the body (e.g. seeing body parts as 'mechanistic spare parts') is related to other values (e.g. liberty, justice, self-development). I hereby distinguish between normativity (prescriptive; as rational ethical justification) and morality (descriptive; an analysis of values and socio-cultural attitudes in a group of people or society of what is right and wrong) (see [33]).

Within the naturalist and constructivist positions, the modification of the body relates to the normative framework of personal interests, social obligations or reciprocal relational structures as determined for instance by post-conventional or feminist views. Of all three, the "transformation"-position is most supportive of the independent development of the idea of the body as an essentially 'unavailable' entity, with a specific inherent value. The terms 'unavailable entity' and 'unavailability' are used as *termini technici *to characterize normative limitations with respect to the body. The body must not be objectified and is never totally disposable for instrumentalisation. This value in its own right nevertheless needs to enter into some kind of relation with other norms and values, in order for positions to be taken from a bioethical perspective on the various forms of modification of the body. According to a rather common point of view, whether an intervention into bodily intactness is morally permissible depends on the agreement of the person in question to suffer such an operation, be it the removal of a kidney or an artistic act as described in the introduction.

I thus want to focus on and discuss the relation of autonomy (as one prominent value in bioethics) and embodiment in the next step. This relationship is, in my opinion, a crucial issue, precisely because one cannot understand the various positions about the body and its meaning for the self without some consideration of liberalism, social conformism and the question of when a human act or decision is authentic, free and autonomous.

### Part 2: More than one relation: embodiment and autonomy

Let me give a short overview of two philosophers who have specifically investigated the relationship of embodiment and autonomy, by criticizing (radical) liberal tendencies in bioethics.

According to Richard Shusterman [34], recent forms of conformism as well as individualism encourage 'somatisation', that is, practical attention paid to the body (through, for instance, cosmetic surgery, body building, medical operations, and piercing). There is an explanation for the positive co-existence of both the cult and the negation of the body: Both trends are rooted in disrespect for the body, as concentration on the mere exterior materiality of the body does not acknowledge the body's independence. The body is no longer perceived as a given fate, but as raw material at the disposal of individual creativity. There are, however, some indications as to the dialectics of these practices: at least in their beginnings, many aesthetic body techniques such as tattooing or piercing can be seen as an individual's expression of resistance against standards and societal body norms. Similar to sports, they can also represent a positive body experience which is obtained through pain. Modifications of the body apparently promise liberties, yet at the same time there is a fear of the enslavement of the body. Shusterman distinguishes a 'somatic of presentation' (a manipulation of outward appearance) and a 'somatic of experience' (new breathing techniques, psychotherapy etc.). With Shusterman, especially the former is criticized. This was due to a critical attitude which interprets attention paid to the body as an already alienated interest in an outward representation, which would therefore inevitably serve the corrupt aims of advertising and propaganda [34]. In contrast, Shusterman recognizes the somatic of the experience as an option that can have constitutive potential for identity and harmony.

In libertarian ethics, 'autonomy' is, as already shown, the normative touchstone for many kinds of body modification. However, as Christman has shown in depth, there are various conceptions and interpretations of autonomy ([35,36,36]). In general terms, autonomy is seen as a basic condition for liberty [37]. Following Carter, the crucial question is – in order to distinguish between 'negative' and 'positive' liberty – whether someone is primarily interested in the degree of external interferences and controls (such as the state, other persons) (=negative liberty) or whether someone advocates the importance of internal factors (such as self-commitments or shared social opinions; = positive liberty). Both ideas of liberty focus attention on the way desires and interest are formed and put into practice [38], while the *content *is not considered (see also [37]. This observation points precisely to the debate over 'bodily autonomy', a term coined by Catriona Mackenzie [39]. Mackenzie develops an account of the theoretical relationships between choice, bodily capacities and autonomy in order to discuss the content of arguments concerning wishes and acts that interfere with embodiment and body modifications. She criticizes the notion of maximal libertarian autonomy that underpins the expansion of available body modifications, rights for body property and the instrumentalisation of the body for personal autonomy [39] because she rejects the idea that maximizing choices automatically increases a person's autonomy. In addition, she rejects the straight liberal maximal choice conception because it provides no normative criteria to assess which choices are autonomy-enhancing and which are impairing. Here, Mackenzie seems to refer to a radical libertarian interpretation of 'liberal ethics', while there are liberal ethicists, most prominently John Rawls [40], who also see self-restriction, fairness and paternalism as parts of a reasonable social morality and as protecting us from unreasonable first-order wishes which endanger our second-order wishes. Referring to a 'relational conception of autonomy', and following Ricoeur's phenomenological approach, namely that human corporeality is the invariant condition of human selfhood, Mackenzie suggests understanding the body as part of our identity, such that her favored notion of bodily autonomy – also as a normative theory – always implies critical reflection on changes of bodily integrity and accepting the "givens of human embodiment" [39] (p. 433).

The attraction of Mackenzie's idea lies in its productive critique of a 'radical' libertarian conception of bodily autonomy, as described above. It helps to detect the weak point of under-complex premises regarding the meaning and condition of the human body as an instrumental means. But again, her ambitious conception of bodily autonomy is itself built upon normative and anthropological premises which are taken for granted. Instead of the phenomenological position she seems to take as given, I would suggest that normative reflections on bodily autonomy should be grounded on several premises, including non-phenomenological positions, as they are also prominent in medical practice or radical postmodern thinking.

What follows for my argumentation? Not only conventional liberal bioethics, but also the critiques of these positions are in need of clarification and justification of their premises regarding embodiment. I understand this critique as a fruitful staring point for re-thinking our initial problem: How do we interpret and conceptualize the body in bioethics? And, secondly, the current international discourse in bioethics has to acknowledge its own diversity in its use of 'body conceptions'. It is scientifically unsatisfactory to 'stick' to some views and reject others as 'ideologies'. The several, conflicting assumptions of what the human body 'is' may result in conflicting ethical judgments. The aim is not so much to overcome all conflicts, but rather to have an explicit discussion on what the body means to bioethics and not only in theoretical papers but also in applied problem-solving ethics. Therefore, I suggest an analytical matrix which allows a self-critical test of various premises by way of a dialectical composition of the various views. It is built upon the idea of a critical reflection of normative and anthropological premises by contrasting them with alternative or even antagonistic conceptions of body-autonomy-relationships. This multidimensional approach functions as a heuristic tool to "identify and test" bioethical assumptions with respect to different epistemic and anthropological premises regarding the body. At the same time the approach sustains tension between different notions of autonomy.

To achieve this, I start from a summary of the two polarized main lines concerning the interpretation of 'autonomy'. According to Christman ([35,36]), 'autonomy' refers, on the one hand, to the potential or actual capability of a person to act and decide independently of external influence and power. This avenue is often stressed in liberal argumentation, where *self-determination *is conceptualized without considering the social influence on norms and preferences. Its main pursuit would be 'negative liberty' (see above). I prefer to talk of self-determination in this understanding of autonomy, because the term stresses the "self" – instead of external determination. On the other hand, 'autonomy' could mean the moral ideal of developing values and normative standpoints by means of criteria of role distance, self-limitation and universalization. This avenue leads in the direction of the above mentioned 'relational autonomy' in which social responsibility and social relationships are the main entrance into understanding autonomy. I call it here 'moral autonomy' to stress the capacity for moral self-reflection, as Mackenzie (p. 429) does: Moral (bodily) autonomy connects to the imagining of the good life through critical and creative self-reflection and the integration of biological, socio-cultural and biographical dimensions of bodily self-representation. This notion of autonomy also appears to be linked to the idea of a successful establishment of a coherent identity, despite fashionable trends and socio-cultural conformity. Both conceptions of autonomy do not necessarily preclude each other from a normative point of view. While the liberal 'minimum' conception of autonomy is understood as a capability to develop and formulate individual preferences despite or precisely because of social influences, the second 'maximum' one is understood as the capability to question and prioritize one's own preferences and to use them for orientation with respect of others' interests and needs.

According to my analysis in part 1, there are also at least two main polarized perspectives on the body. These also may enrich the bioethical debate. On the one hand, there is the phenomenological perspective which combines both the perception of the individual's material and anthropological limitations (the physical dimension) and the lived-body phenomena such as sensual perceptions or pain. The other view understands embodiment as a textually or culturally inscribed 'exterior' (such as categorisation into specific aesthetic, social, gender or medical-scientific 'classes' would be). As a result, we now have four different specific perspectives for the relationship. Each is based upon one of the specific interpretations of embodiment and autonomy that I have worked out above. The four perspectives are not hierarchically understood; one could 'start' reading the matrix from the conventional, "liberal-materialistic" perspective and end at the "communitarian-deconstructivistic" position, but also vice versa (I will illustrate this for concrete cases in the next section). But in order to avoid misunderstandings, I choose more adequate labels to describe these four perspectives:

#### I) "Bodily self-determination"

Autonomy as the right to bodily self-determination refers in this view to the defense of one's own body against direct and indirect interventions by third parties. The body represents the immediate access to one's own personality (i.e. to 'express' one's own opinion by a body modification), and at the same time can be regarded materialistically as a transformable entity. Autonomy remains fixed to the somatic/bodily- conveyed capabilities of personal identity (i.e. to communication, to coping with pain, to the conscious realization of personal characteristics). However, this view does not have to lead to the conclusion that an instrumentalisation of the body always means an instrumentalisation of the person, provided that the interventions in question are agreed to by this person, and the natural basis for this person's identity remains intact.

#### II) "Respect for the bodily unavailability of the other"

Moral autonomy, as part of the self-restriction that I have discussed above, includes the respect for another person's bodily integrity, even if it conflicts with one's own preferences and aims of action. This necessitates a critical reflection on the ways in which one deals with the body of others and one's own within the social and cultural space. This respect is more than a negative right to repel claims of others. It understands respect for others as reciprocity for the wish to be respected. First and foremost, this respect for the bodily unavailability of others allows critical reflection of own needs for other bodies. But it also includes considerations about one's own body images, bodily integrity and desires for body modifications. Can one rightfully deduce certain demands to maintain or form bodily integrity on this basis, particularly so if this has implications for third parties? A self-critical view on body images and ideals could be required if one cannot be sufficiently sure of avoiding implicit or explicit discrimination of those who diverge from this ideal. In addition, moral autonomy can involve taking political-normative initiative for the bodily integrity of others even if one is not affected personally. This could be done through advocacy, esp. for those who cannot articulate their own interests and views. This includes the commitment to political discussions about the unavailability of the body of third parties.

#### III) "Care for bodily individuality"

Autonomy as self-critical reflection includes the fulfillment of individual interpretations of what a good life is for me, including a form of care and concern for my body. Therefore, an understanding of the body can be regarded as part of a conception of the good life: one's own interests and desires are linked to imaginations of bodily perception and expression, and to bodily-mediated actions such as communication, love and sensations. Visions of the good life include the striving for aesthetic values, and the development and stabilization of an identity as conveyed through sexuality, appearance as bodily characteristic and bodily techniques (such as in the acts of eating or moving etc.). Such bodily features are always situated within a complex understanding of individual normality, of political and social standardization, and of historical and cultural difference. Embodiment is critically investigated as socially constructed and discursively negotiable. This emphasizes and makes comprehensible the role of individual care for bodily characteristics: the central normative element of this care may well be the recognition of a difference rather than of a norm or normality. Care for bodily individuality goes further than having a maximal choice for body modification: It also includes the idea that the self and personhood are built upon individual appearance and individual body language and styling. Care includes the protection of bodily individuality by maintaining one's own identity even if one's body appears different and 'strange' to others. Recognition of bodily individuality could be understood as recognition of being different (to others). Therefore this need not lead to an exclusion of the other but can support the fortification of one's own self-determination through dialogue and a creative approach to the other. The background to this is the idea that there exist several ways of dealing with embodiment (and its weaknesses) which serve as sources of ideas for the conduct of life, and hence of ideas of the good life.

#### IV) "Recognition of bodily cooperation"

Autonomy as the opportunity and capacity to develop a self as an autonomous person may at the very least require the right to one's own social identity within the framework of group membership. This group is assumed to express itself by means of specific forms of embodiment, of bodily interaction and of bodily-constituted communities; and to build a harmonic cooperation, here called 'bodily cooperation'. This recognition is built upon the care for bodily individuality but refers to forms of bodily expression in which the body is constitutive for specific social interactions. The building of stable social relationship such as parenthood, partnerships or friendship could not easily be thought without having bodily contact through touching or sharing bodily experience. For instance, most forms of sexuality are constitutively bodily social interactions, as are maternity or the social handling of dead human beings. Recognition of such bodily interaction leads to political and social recognition of those communities which are different in sexual preferences, e.g. homosexual communities.

## Discussion: Increasing sensitivity for various normative perspectives of the body

What conclusions can be drawn once we open up these four perspectives instead of the common narrowing to only one favored perspective? In the last section, I want to use the approach developed in part 2 for an improved, premise-critical description of ethical problems in recent biomedicine. The chosen examples of issues in transplantation medicine, neuroprosthesis or cosmetic surgery present serious cases of 'transgressing borders'. Because of limitations of space, I will restrict the final discussion to the aim of showing the increased sensitivity to the various perspectives of normative judgments through different ways of formulating the starting problem.

I therefore start again from a liberal conception in which the argument of *bodily self-determination *is vehemently used to justify the right to body modification as long as informed consent is given by the affected person. As long as biomedical technologies are perceived as means to achieve emancipation from bodily limitations (through illness, pain or death) their legitimacy does not seem to be in doubt. But it is decisive whether new options secure, impair or increase the preservation of a person's interests and autonomy through body modifications. For instance in the case of the transplantation of organs, how is the freedom of a patient to chose between options as provided through the medical system safeguarded within the framework of information, availability of medical treatments and agreement procedures? A special case is the possible commercialization of the donation of organs. The argument of bodily self-determination seems to support a liberalization of the trade in organs as long as it is guaranteed that possible medical risks are reliably assessed and made clear to the agent, and that injustice through possible exploitation is avoided [41]. But in the same field, we have to consider the understanding of what constitutes a person, as exemplified by the question whether self-determination continues beyond a person's heart death or total or part brain death. Here, the anticipated relationship of self-determination and embodiment (in the sense of an understanding of the body that has to be interpreted individually) is decisive. Is it a truly personal affair to decide on what should happen to one's dead body, or does one need to respect the piety of relatives and therefore accept certain limits? The former distinction between human being and machine, mind and body, is blurred by the technology itself (as 'dead' persons are kept 'quasi-alive' by heart-lung-machines). If a person's autonomy is linked only to current bodily self-determination and constructs the body as a physical instrument, further- reaching claims such as the social obligation to help other patients with organs eventually succeed, even if the dead person refused the explantation of organs whilst alive.

But further interesting perspectives appear when bodily self-determination is complemented with concerns about the respect for the bodily unavailability of another person. The constructed case of an individual decision-making process often cuts out medical and social reality: This includes questions about the person who serves as an organ donor or who carries out an operation, for instance as a doctor. For example, in the case of a living donation one needs to raise the question as to whether the living donor of a kidney considers the act of donation as a voluntary, autonomous decision, but also whether the potential organ receiver has a right to ask for the donation. The transgression of the internal-external-borders ("My kidney in your body?") opens the new field for the moral assessment of identity and bodily integrity. Taking seriously the *moral respect for others' bodily integrity *opens as such a new perspective: Doctors, as well as any potential recipient of the organ have to acknowledge the moral dimension of their decision (to conduct the operation, and to receive the organ) and to take responsibility for their respective roles within the decision-making process. The critical reflection on the respect for the bodily integrity of the other also allows for a consideration of the impacts of modern biomedicine on people who are not directly affected, in terms of a possible discrimination of third parties. Discriminating with regard to others means to disadvantage them on the basis of their membership in a specific group although this specificity does not justify such inequality in treatment. While the focus on bodily self-determination neglects the dimension of future social developments, the respect for bodily unavailability opens the door for critical social impacts, even if they are indirect and only a future possibility. Such problems could be approached by considering slippery-slope arguments. Cosmetic surgery could hereby be seen as challenge to the border between what is seen as natural and what is artificial. While this 'border' is not said to be a distinction between morally right or wrong, its transgression implies important questions of authenticity and cultural standards. For instance cosmetic surgery on an adult woman very often seems to be legitimated with reference to bodily self-determination. Taking influential pop cultural shifts in body images as possible and likely, this may result in a successive, implicit social compulsion for next generations to undergo similar modifications of the body. The moral dimension of such individually legitimate decisions unveils ethical problems for those who are rather dependent on cultural standards (such as adolescents).

The conception of self-determination is related to a conception of the good life insofar as the fulfilling of single preferences and second-order interests could be seen as embedded in the 'whole' perspective of what a person should be, of what is part of his/her self and identity. The stabilizing effect of the exclusion of and separation from others on personal identity should not be underestimated [42]. This new idea of individual care leads to a positive effect that stabilizes identity. The role of *care for bodily individuality and its characteristics*, for the development of identity and thus for self-determination is further supported by the process of individualization and the deconstruction of fixed socially constructed categories such as 'healthy' and 'ill', 'ugly' and 'beautiful' or 'natural' and 'artificial'. For instance, it allows a more open discussion of how to assess neuroprosthesis and brain implants to cope with certain disabilities (such as deafness), Parkinson disease (e.g. treated with xenotransplants) or patients with the Tourette-syndrom (a disorder, which is characterized by uncontrollable vocalizations and movement and treated with deep brain stimulation) (see e.g. [43]). These biomedical technologies could question the 'border' between human being and machine or animal. If the body is seen only as a material basis or as depending on individual perception, the bioethical discourse is then poised on the (empirical) question of whether the prostheses or xenotransplants are able to change 'personal identity'. Instead, realizing that the border itself is questionable, on the one hand, and that normative recognition of bodily cooperation, on the other hand, may count, alternative solutions such as the improvement of care and the reduction of barriers on the social, structural or town-planning for elderly or mentally ill people will be seriously discussed. Additionally, this directly opens up issues of distributive justice: We have to face the problem that excessive use of biomedical solutions could forget all those disabled people and patients who – for personal or structural reasons – do not have access to biomedicine.

Finally, the consideration of the *recognition of bodily-constituted communities and bodily cooperation *allows us to question whether some biomedical practices could destroy cultural identities, for instance as signified by a loss of sign language due to the use of cochlea implants [44], or whether it also contributes to the gestation of new ones (through the development of new collectives of patients, for instance). The normative tension between bodily self-determination and care for one's bodily individuality gives rise to a discourse over the extent to which the acceptance or refusal of an intervention into the body is rooted in a comprehensible insecurity or desired unavailability with respect to one's own body. Adding the perspective of the possible value of bodily-constituted communities allows new forms of assessing social actions and communication. Since the debate about race, sexuality, ethnicity and disability, we have seen by way of the negative effects of embodiment its crucial role in the perception of other 'cultural' identities. For instance, the social and political dimension of patients' self-help groups could be better discussed as part of a socio-political dimension in the medical system than by focusing on individual decision-making. Patients support and advise each other; they share something which not only separates them from others, but also strengthens them: the existential experience of illness or of the long process of therapy and recovery [45].

## Conclusions: The loss of self-evident truths

The opening for various relationships between autonomy and embodiment provides a central interface for the ethical reflection about who can decide when and how about one's own body. What elements of a person can be regarded as available or unavailable at which points in time during the process of this person's life or dying? Some liberal ethicists criticize the 'body boom' in ethics as a "neo-heathen body cult" [46], because they view it as inappropriate to refer to the body as morally relevant. However, as I argued above, this assumption could be self-contradictory if proponents of the liberal conception of self-determination recognize the principle of non-maleficience as a moral duty to act in a responsible way – as many scholars do. Non-maleficience and the obligation to reduce suffering are linked to a specific concept of the body – a body which is able to 'suffer' and 'feel pain' and can be 'harmed'. Instead of neglecting one's own anthropological and epistemic premises about this suffering body I suggest to be aware of them. I conclude therefore that the bioethical procedure of detecting and describing ethical dilemmas should also take into account the ways and limits of perceiving one's own body and those of others. From here, it should not be concluded that any kind of biotechnology is morally problematic just because it annihilates 'difference' (for instance through the idea of making an 'ill' person 'healthy') nor is it generally justified just because patients gave their 'free' informed consent.

The body is a challenge for bioethics, because autonomy as the idea of the 'unavailability' of the body relies on various premises regarding the manner in which cultural and personal identity is built upon bodily practices, bodily constitutions and body images. Within the liberal bioethical context, bodily self-determination is often understood as a minimal moral consensus based on a legitimate resistance against medical (or state) paternalism. But as I showed so far, bioethics provides more than insisting on this minimal consensus; ethical reflection also serves a fruitful idea of a reflective self-relation of the moral agent. This reflection makes it necessary to think about the normative meaning of specific bodily related interactions with others and the respect and care for others' bodily integrity.

However, the categories for the cultural and natural order of the body as described above are not regarded as having a moral value in their own right, but as being very value-laden. Thus, the suggested matrix is open to various interpretations and offers both linguistic and argumentative access to critical inquiry and to the different ways of being a lived body or a thing body [30]. One should also note that the loss of order, as I described the moral and epistemic uncertainties towards human bodies in Part 1, is constantly discussed within the bioethical debate, but is labeled in a different way: as the conflict about the so-called 'moral status' of various entities, e.g. of a human embryo, of animals, or of brain-dead persons.

The loss of self-evident truths may often be regarded as a specifically modern phenomenon or even as the tragedy of modernity. Particularly the wider bioethical perspective shows to which extent epistemological and normative views are intertwined. However, in the course of self-reflection this loss can also be seen as something positive: as an opportunity for self-re-interpretations.

## Competing interests

The author declares that she has no competing  interests.  
